# Activation of cGAS–STING axis by CO@OMVs promote macrophage mitophagy for CIRI treatment

**DOI:** 10.1063/5.0345675

**Published:** 2026-09-28

**Authors:** Yiwei Chen, Shuntong Hu, Juan Huang, Pangyao Long, Jiahao Zhou, Longfeng Ran, Jinze Wu, Xiaobo Li, Huaizheng Liu, Yi Yuan, Dujie Xie

**Affiliations:** 1 Department of Neurology, The Third Xiangya Hospital, Central South University, Changsha 410013, China; 2 State Key Laboratory of Quality Research in Chinese Medicine, Institute of Chinese Medical Sciences, University of Macau, Taipa 553000, Macau, China; 3 Department of Neurology, Chengdu First People's Hospital, Chengdu 610041, China; 4 Department of Emergency, The Third Xiangya Hospital, Central South University, Changsha 410013, China

## Abstract

This study investigates the effects of nanobody-engineered bacterial outer membrane vesicles (CO@OMVs) on cGAS–STING-associated signaling, macrophage mitophagy, and cerebral ischemia-reperfusion injury (CIRI). Following extraction and characterization of CO@OMVs, *in vitro* experiments in RAW 264.7 macrophages demonstrated increased levels of cGAS–STING-associated signaling molecules, enhanced mitophagy, increased tumor necrosis factor-α (TNF-α) secretion, and decreased IL-6 secretion. In a CIRI mouse model, CO@OMVs reduced local inflammatory markers and apoptosis and improved cognitive and neurobehavioral functions. Multi-omics analyses further supported the therapeutic potential of CO@OMVs in mitigating early brain injury.

## INTRODUCTION

Cerebral ischemia-reperfusion injury (CIRI) is secondary damage that occurs in brain tissue during the restoration of blood supply following a stroke, often leading to severe neurological dysfunction and high mortality.^1,2^ The pathogenesis of CIRI is multifactorial, involving excessive oxidative damage, neuroinflammatory activation, programmed cell death, and impairment of blood–brain barrier integrity.^3–5^ Although various therapeutic approaches, such as anticoagulants, antioxidants, and anti-inflammatory drugs, have been developed, their effectiveness is limited, and they are unable to effectively prevent or reverse the neurological damage caused by CIRI.^6^ Thus, identifying innovative therapeutic approaches is essential for alleviating brain injury caused by CIRI and improving patient survival and quality of life.

Macrophages are important modulators of the occurrence and progression of CIRI.^7^ As major components of innate immunity, macrophages may aggravate neuroinflammation by releasing pro-inflammatory cytokines, while under certain circumstances, they can also contribute to tissue remodeling and repair via regulatory signaling pathways.^8–10^ The involvement of macrophages in CIRI is closely associated with activation of the cyclic GMP-AMP synthase (cGAS)–stimulator of interferon genes (STING) axis. This axis acts as a DNA-sensing pathway that can recognize mitochondrial DNA released by damaged cells, thereby initiating antiviral immune responses and inflammatory reactions.^11,12^ Additionally, mitophagy, a form of cellular autophagy, can alleviate oxidative stress and inflammation within cells by clearing damaged mitochondria.^13^ Therefore, regulating macrophage mitophagy, particularly through the cGAS–STING axis, could be an effective strategy for mitigating CIRI-related damage.

Outer membrane vesicles (OMVs) are nanoscale vesicles released by Gram-negative bacteria, characterized by high biocompatibility and immunomodulatory capabilities.^14,15^ In recent years, OMVs have shown broad application potential in fields such as immunotherapy, vaccine development, and drug delivery.^16–18^ Through genetic engineering, OMVs can be designed as specific targeting tools, carrying particular antigens or drug molecules to enhance their therapeutic effects.^19–21^ Nanobodies, a class of small antibodies derived from camelids, have shown promising applications in the treatment of various diseases due to their high stability and strong targeting ability.^22^ Combining nanobodies with OMVs can further enhance the targeting specificity and biological activity of OMVs, thereby improving their application in disease treatment.^20,23^

The cGAS–STING axis, as a key immunoregulatory pathway in CIRI, has been a research focus in recent years. Targeting the cGAS–STING axis can significantly regulate macrophage function, reduce inflammatory responses, and promote tissue repair.^12,13^ Nanobody-engineered bacterial OMVs (CO@OMVs) combine the high targeting specificity of nanobodies with the biological activity of OMVs, modulating cGAS–STING-associated signaling and promoting macrophage mitophagy, thereby modulating immune function. This strategy not only offers a new therapeutic approach but also provides insights into the pathological mechanisms of CIRI.

The present study explores how CO@OMVs regulate the cGAS–STING signaling pathway, promote macrophage mitophagy, and ultimately mitigate CIRI. A key novelty of this work is the integration of nanobodies with OMVs, with the cGAS–STING axis selected as the central therapeutic target, thereby providing a potential nanotechnology-based intervention strategy. Both *in vitro* and *in vivo* studies demonstrated that CO@OMVs significantly alleviated inflammation and apoptosis following CIRI, thereby improving neurological functional recovery. Furthermore, rescue assays demonstrated that the cGAS–STING pathway is required for the protective effects observed. Overall, this study offers new mechanistic insights into CIRI treatment and expands the potential application of nanotechnology in neural injury therapy, with important scientific and clinical relevance.

## METHODS

### Nanobody engineering and bacterial culture

The coding sequence of the cGAS-specific nanobody, together with the sequence encoding the bacterial outer membrane protein OmpA, was inserted into the pET28a plasmid vector (Genewiz, Suzhou, China). The resulting recombinant construct was subsequently introduced into *Escherichia coli* BL21(DE3) cells [Tiangen Biotech (Beijing) Co., Ltd., China] with a wild-type lipopolysaccharide (LPS) background for OMV production. The engineered bacterial strains were grown in LB medium at 37 °C with shaking at 180 rpm until the OD600 reached 0.6. Protein expression was subsequently induced by adding isopropyl β-D-1-thiogalactopyranoside (IPTG, I6758, Sigma-Aldrich, USA), followed by incubation at 16 °C with shaking at 160 rpm for an additional 14 h. When necessary, kanamycin or ampicillin was added to the medium at final concentrations of 50 *μ*g/ml.

### OMVs extraction

After an additional 14 h of shaking culture, the *Escherichia coli* BL21(DE3) suspension was centrifuged at 10 000 × g for 10 min at 4 °C using a Sorvall ST16R centrifuge (Thermo Scientific) to eliminate bacterial cells. The collected supernatant was passed through a 0.45 *μ*m filter (Vacuum Filtration System, Corning) and subsequently concentrated with a 100 kDa molecular weight cutoff (MWCO) centrifugal filter device (Millipore). The concentrated sample was further filtered through a 0.22 *μ*m membrane filter (Millipore) to remove residual bacteria and debris. Subsequently, the filtrate was ultracentrifuged at 150 000 × g for 3 h at 4 °C using an XPN-100 ultracentrifuge (Beckman). After removal of the supernatant, the obtained pellet was resuspended in PBS and stored at −80 °C. The protein concentration of CO@OMVs was determined using a Micro BCA Protein Assay Kit (Pierce, USA). Fusion protein expression was confirmed by sodium dodecyl sulfate-polyacrylamide gel electrophoresis (SDS-PAGE) and Western blot (WB) analysis.

### Characterization of OMVs

The morphology and particle size of OMVs and CO@OMVs were evaluated by transmission electron microscopy (TEM; JEM-1230, JEOL, Japan) and nanoparticle size analysis using a NanoSight NS300 instrument (Malvern Panalytical, UK). For TEM analysis, samples were loaded onto Formvar carbon-coated grids for 2 min, negatively stained with 2% uranyl acetate, dried at room temperature, and observed with a TEM at 80 kV. Vesicle homogeneity among different sample groups was assessed with software from Soft Imaging System (Switzerland), and vesicle diameters were calculated accordingly. The size distribution and polydispersity index (PDI) of OMVs and CO@OMVs suspended in PBS at a final concentration of 50 *μ*g/ml were determined by dynamic light scattering (DLS). The Z-average particle size was used to indicate the average vesicle diameter, whereas the PDI reflected the breadth of size distribution and the degree of uniformity or heterogeneity.

The expression of the His-tagged nanobody fusion protein on CO@OMVs was confirmed by WB analysis. Total bacterial proteins were isolated using a bacterial protein extraction kit (Catalog No. C600596, Beijing Puyi Biotechnology Co., Ltd., China). Briefly, bacterial cells were collected by centrifugation at 5000 × g for 10 min at 4 °C and washed thoroughly. The cell pellet was then resuspended in lysis buffer containing phenylmethylsulfonyl fluoride (PMSF; Solarbio, China; 4 *μ*l/ml bacterial culture) and lysozyme (80 *μ*l/ml bacterial culture), followed by incubation at 37 °C for 30 min. To promote complete cell disruption, the suspension was shaken for an additional 10 min. DNase I/RNase solution was then added at 20 *μ*l per sample, and the mixture was incubated with shaking at 37 °C for another 10 min. Insoluble debris was removed by centrifugation at 3000 × g for 30 min at 4 °C, and the supernatant was collected for subsequent analysis. For protein preparation from CO@OMVs, the vesicles were resuspended in radioimmunoprecipitation assay (RIPA) buffer supplemented with 1 mM PMSF (Solarbio, China) and lysed for 15 min. After centrifugation at 13 000 × g for 15 min, the supernatant was collected. Protein samples were separated on a 10% SDS-PAGE gel and transferred to a polyvinylidene fluoride membrane. After blocking with 10% non-fat milk, the membrane was incubated with an anti-His-tag antibody for 2 h at room temperature. The membrane was then washed three times with Tris-buffered saline containing 0.5% Tween 20 (Solarbio, China), followed by incubation with an horseradish peroxidase (HRP)-conjugated secondary antibody for 1 h at room temperature. Immunoreactive bands were detected using SuperSignal West Pico chemiluminescent substrate (Thermo Scientific, Rockford, USA).

### Stability and functional testing

To evaluate the storage stability of CO@OMVs, samples were stored at 4 °C or −80 °C and collected at 0, 1, 2, 4, and 8 weeks. At each time point, the particle size distribution and concentration of CO@OMVs were measured by nanoparticle tracking analysis (NTA) using a NanoSight NS300 instrument (Malvern Panalytical, UK). Briefly, samples were appropriately diluted, loaded into the sample chamber, and subjected to data acquisition and analysis. All measurements were performed in triplicate, and the mean values were calculated.

To assess the binding specificity of CO@OMVs to cGAS, an enzyme-linked immunosorbent assay (ELISA) was performed. Briefly, 96-well plates were coated with recombinant cGAS protein and then incubated with CO@OMVs at 10 *μ*g/ml, with 100 *μ*l added to each well, at 25 °C for 2 h. Subsequently, 100 *μ*l of HRP-conjugated anti-His-tag antibody diluted 1:6000 in PBST was added to detect CO@OMVs. After three washes with PBST, the immune response was visualized using the HRP substrate 3,3′,5,5′-tetramethylbenzidine (TMB; Abcam, UK). Absorbance was then recorded at 450 nm to determine the binding capacity of CO@OMVs to cGAS.

### Cell culture

The murine macrophage cell line RAW 264.7 was purchased from the American Type Culture Collection (ATCC, USA). Cells were seeded in culture flasks and maintained in RPMI 1640 medium (R999297, MACKLIN, Shanghai, China) containing 10% (v/v) FCS, L-glutamine, penicillin-streptomycin, sodium pyruvate, and 4-(2-hydroxyethyl)-1-piperazineethanesulfonic acid (HEPES), with the pH adjusted to 7.2–7.5.

The murine 4T1 breast cancer cell line was likewise sourced from ATCC. 4T1 cells were seeded in culture flasks and maintained in DMEM (D1145, Merck, Germany) supplemented with 10% fetal bovine serum (FBS) and 1% antibiotics. Cells were cultured in a humidified incubator at 37 °C with 5% CO_2_.

### OMVs treatment

To evaluate macrophage-mediated phagocytosis of tumor cells, 4T1 murine breast cancer cells (ATCC, USA) were pretreated with PBS, OMVs (10 *μ*g/ml) or CO@OMVs (10 *μ*g/ml) at 4 °C for 2 h. The pretreated 4T1 cells were then co-cultured with RAW 264.7 murine macrophages (ATCC, USA) at a macrophage-to-tumor cell ratio of 1:2 at 37 °C for the indicated durations. Macrophage phagocytic activity against 4T1 cells was assessed by flow cytometry and quantified as the proportion of GFP-positive cells within the F4/80-positive macrophage population. For fluorescence imaging, RAW 264.7 cells were labeled with DiI (2 *μ*M; Beyotime, China; Cat. #C1036) and co-cultured with pretreated 4T1 cells for 2 h. After washing twice with PBS, the cells were observed by laser scanning confocal microscopy (LSCM). For Giemsa staining, RAW 264.7 and 4T1 cells were processed using the same co-culture procedure. The cells were fixed with 4% paraformaldehyde for 20 min, washed three times with PBS, stained with Giemsa solution (Solarbio, China; Cat. #G1010) at room temperature for 10 min, and imaged under a light microscope.

### CCK-8 assay

Cell proliferation was assessed using the Cell Counting Kit-8 (CCK-8, Dojindo, Japan). All cell cultures were performed in DMEM containing 10% FBS (Gibco, USA) and 1% penicillin/streptomycin (Gibco, USA) in a humidified incubator at 37 °C with 5% CO_2_. Cells were seeded into 96-well plates at a density of 5 × 10^3^ cells per well, with five replicate wells per group. After overnight attachment, CO@OMVs were added at a final concentration of 100 *μ*g/ml, and the cells were incubated for 24, 48, or 72 h. At each indicated time point, 10 *μ*l of CCK-8 reagent was added to each well, followed by incubation for an additional 2 h. Absorbance was measured at 450 nm using a microplate reader (Multiskan FC, Thermo Fisher Scientific, USA). Control cells were cultured without CO@OMV treatment and processed in parallel.

### Flow cytometry

Cell apoptosis was assessed using an Annexin V-FITC/PI Apoptosis Detection Kit (BD Biosciences, USA). Cells were cultured in DMEM supplemented with 10% FBS and 1% penicillin/streptomycin at 37 °C in a humidified incubator containing 5% CO_2_. Cells were seeded into 6-well plates at a density of 1 × 10^6^ cells per well and allowed to adhere overnight. CO@OMVs were then added at a final concentration of 100 *μ*g/ml according to the experimental design, and cells were incubated for 24, 48, or 72 h. After treatment, cells were washed twice with PBS, detached with 0.25% trypsin, collected, and centrifuged at 300 × g for 5 min. The cell pellets were resuspended in 1× Annexin V binding buffer. Subsequently, 5 *μ*l of Annexin V-FITC and 5 *μ*l of PI were added to each sample, gently mixed, and incubated for 15 min at room temperature in the dark. Stained cells were immediately analyzed using a BD FACSCanto II flow cytometer. At least 1 × 10^4^ cells were acquired for each sample, and FITC and PI signals were recorded. Data were analyzed using FlowJo software (FlowJo, LLC, USA). Cells were classified as viable cells (Annexin V^−^/PI^−^), early apoptotic cells (Annexin V^+^/PI^−^), and late apoptotic or necrotic cells (Annexin V^+^/PI^+^). For apoptosis assessment in the *in vivo* rescue/activation experiment, brain tissue-derived single-cell suspensions were stained using the same Annexin V-FITC/PI protocol and analyzed by flow cytometry.

### RNA sequencing (RNA-seq) analysis

Total RNA was extracted from treated cells using TRIzol reagent (Invitrogen, USA). RNA samples were frozen and sent to Biomarker Technologies (Beijing, China) for whole-transcriptome sequencing. RNA concentration, purity, and integrity were evaluated using a NanoDrop 2000 spectrophotometer (Thermo Fisher Scientific, USA), an Agilent 2100 Bioanalyzer (Agilent Technologies, USA), and a LabChip GX system (Caliper Life Sciences, USA). Only RNA samples meeting the quality requirements were used for library construction and subsequent sequencing on the Illumina NovaSeq 6000 platform. Bioinformatics analysis was performed using BMKCloud. The Mus musculus genome assembly GRCm38/mm10 was used as the reference genome. Gene and transcript expression levels were quantified as FPKM values, representing fragments per kilobase of transcript per × 10^6^ mapped reads.

### Proteomics analysis

Proteins were extracted from treated cells and subsequently quantified using a BCA Protein Quantification Kit (Pierce, USA). Equal amounts of protein samples were labeled following the protocol provided with the iTRAQ labeling kit (AB Sciex, USA). After labeling, the samples were pooled and fractionated by high-performance liquid chromatography (HPLC). The resulting peptide fractions were then analyzed by mass spectrometry using a Q Exactive HF-X mass spectrometer (Thermo Fisher Scientific, USA).

The acquired mass spectrometry data were processed with MaxQuant software for peptide identification and quantitative analysis. Differentially expressed proteins (DEPs) were screened using DESeq2, with the significance threshold set as |log_2_FoldChange| > 1 and *p* < 0.05. Ingenuity pathway analysis (IPA) was subsequently applied to investigate the biological functions and signaling networks associated with these DEPs, particularly those involved in mitophagy, inflammatory regulation, and immune responses.

### CIRI model establishment

*In vivo* animal experiments were conducted to evaluate the mechanism by which CO@OMVs alleviate early brain injury in a CIRI mouse model. The experimental procedure is summarized in Fig. S1. Eight-week-old male C57BL/6 mice from Charles River Laboratories (USA) were used, with 10 mice per group. Animals were housed under standard conditions at 22 ± 2 °C, 50%–60% humidity, and a 12 h light/dark cycle.

For therapeutic assessment, mice were randomly divided into four groups (n = 10 per group): Sham, CIRI Control, OMVs Control, and CO@OMVs treatment. Mice in the Sham group underwent anesthesia and surgery without arterial occlusion. The CIRI Control group was subjected to middle cerebral artery occlusion (MCAO) without treatment. The OMVs Control and CO@OMVs treatment groups received tail-vein injections of OMVs or CO@OMVs, respectively, at 100 *μ*g/kg after CIRI modeling. For the rescue/activation experiment, CIRI model mice were assigned to OMVs treatment, OMVs treatment + 3-MA, OMVs treatment + rapamycin, CO@OMVs treatment, CO@OMVs treatment + 3-MA, and CO@OMVs treatment + rapamycin groups. 3-MA (10 mg/kg, Sigma-Aldrich, USA) was used as a mitophagy inhibitor, and rapamycin was used as an autophagy activator. The OMV and CO@OMV doses were selected based on preliminary dose-optimization data showing optimal efficacy with minimal toxicity at this concentration.

The CIRI model was established using MCAO. After anesthetizing the C57BL/6 mice, the common carotid artery, internal carotid artery, and external carotid artery were exposed and ligated. A small opening was created in the external carotid artery, through which a nylon filament was advanced into the middle cerebral artery to interrupt cerebral blood flow. Occlusion was maintained for 20–60 min, after which the filament was gently removed to initiate reperfusion. The surgical incision was subsequently closed, and postoperative care was performed while neurological deficits and tissue injury were continuously monitored.

Immediately after surgery, mice received tail-vein injections according to the assigned groups. CO@OMV-treated mice received 100 *μ*g/kg CO@OMVs, OMV-treated mice received 100 *μ*g/kg OMVs, and rescue/activation groups received the corresponding vesicle treatment combined with 3-MA or rapamycin. Control mice were administered an equal volume of PBS.

Postoperative behavior changes, weight changes, and survival were observed daily. On day 7 post-surgery, behavioral tests and histological analyses, including hematoxylin and eosin (H&E) staining and immunohistochemistry (IHC) analysis of brain tissue, were conducted to verify successful CIRI model establishment and evaluate the therapeutic effects of CO@OMVs.^24^

### *In vivo* biocompatibility assessment of CO@OMVs

To evaluate the *in vivo* biocompatibility of CO@OMVs,^25^ mice were randomly divided into four groups, each containing six animals with balanced sex distribution: Control, OMVs, low-dose CO@OMVs, and high-dose CO@OMVs. The Control group underwent anesthesia and surgical procedures without basilar artery perforation. The OMV group received a tail-vein injection of extracted OMVs (100 *μ*g/kg). The low-dose and high-dose CO@OMV groups received CO@OMVs by tail-vein injection at 100 and 1000 *μ*g/kg, respectively. Blood samples were collected 24 h after a single injection, and serum ALT (C009-2-1, Nanjing Jiancheng), AST (C010-2-1, Nanjing Jiancheng), CRE (C011-2-1, Nanjing Jiancheng), and BUN (C013-2-1, Nanjing Jiancheng) levels were measured to evaluate hepatic and renal toxicity.

### Morris water maze (MWM) test

The MWM test was performed to evaluate spatial learning and memory in mice. Before the probe trial, mice underwent training for five consecutive days, with two trials per day separated by a 4 h interval. During each training trial, mice were placed into the maze from different starting points and allowed to search for a hidden platform submerged beneath the water surface. Escape latency and swimming trajectories were recorded, and the mean escape latency and swimming distance were calculated after each training day. On day 6, the hidden platform was removed, and a probe trial was conducted. The time spent in the target quadrant where the platform had previously been located, as well as swimming paths, was recorded. Behavioral data were acquired and analyzed using the Smart Video Tracking System (Panlab Harvard Apparatus, Spain). Spatial learning and memory performance were assessed based on escape latency, swimming distance, and probe trial parameters.

### Rotarod test

Motor coordination was assessed using the rotarod test. Briefly, each mouse was placed on a rotating rod initially set at 5 rpm, which was then accelerated by 2 rpm per minute. The latency to fall, defined as the time each mouse remained on the rod before falling, was recorded. Each mouse completed three trials with a 15 min interval between trials, and the average latency to fall was used for analysis. The test was performed using a Rotarod System (Ugo Basile, Italy).

### H&E staining and immunohistochemistry (IHC)

After completion of the behavioral assessments, mice were anesthetized with isoflurane. Once a deep anesthetic state was achieved, cardiac perfusion was performed with PBS to remove blood, followed by brain fixation with 4% paraformaldehyde. The brain tissues were subsequently harvested and immersed in 4% paraformaldehyde at room temperature for 24 h for further fixation.

Brain sections were then processed for routine H&E staining. Briefly, paraffin-embedded sections were deparaffinized in xylene twice for 10 min each and rehydrated through a graded ethanol series of 100%, 95%, 85%, and 70% ethanol for 5 min each, followed by rinsing with distilled water. The sections were stained with hematoxylin for 5 min, washed under running tap water for 10 min, differentiated in 1% acid alcohol for 5 s, rinsed again with tap water, blued in 0.5% ammonia water for 1 min, and washed thoroughly. Subsequently, the sections were stained with eosin for 2 min and washed under running tap water for 5 min. Finally, the sections were dehydrated through graded ethanol solutions of 70%, 85%, 95%, and 100% for 5 min each, cleared twice in xylene for 5 min each, and mounted with neutral balsam.

IHC analysis was performed using anti-TNF-α (Abcam, UK, Cat# ab1793), anti-IL-1β (Abcam, UK, Cat# ab9722), anti-LC3 (Cell Signaling Technology, USA, Cat# 3868S), and anti-p62 (Abcam, UK, Cat# ab56416) antibodies. Briefly, tissue sections were deparaffinized in xylene, rehydrated through a graded ethanol series, and subjected to antigen retrieval in citrate buffer (pH 6.0) by microwave heating. After boiling, the sections were maintained at high temperature for 15 min and then cooled to room temperature. To minimize nonspecific staining, sections were blocked with 5% bovine serum albumin (BSA; Sigma-Aldrich, USA, Cat. #A2153) for 30 min at room temperature. The sections were then incubated overnight at 4 °C with primary antibodies against TNF-α, IL-1β, LC3, and p62, each diluted 1:200. After three washes with PBS, HRP-conjugated secondary antibodies (Santa Cruz Biotechnology, USA, Cat. #sc-2004; 1:500) were applied and incubated for 1 h at room temperature. Color development was performed using a DAB substrate kit (Thermo Fisher Scientific, USA, Cat. #34002), with the reaction time controlled according to staining intensity, generally within 1–3 min. The sections were counterstained with hematoxylin, dehydrated through graded ethanol, cleared in xylene, and mounted with neutral balsam. Images were captured using a Leica DM750 optical microscope. For each sample, at least five fields were examined, and representative images were selected for analysis.

### Assessment of cGAS–STING-associated signaling molecules

Total protein was extracted from treated cells and harvested brain tissues using RIPA lysis buffer (Beyotime, China, Cat. #P0013B). Briefly, samples were lysed on ice for 30 min and further homogenized using an ultrasonic homogenizer (Sonics Vibra-Cell, Sonics & Materials, Inc., USA) at 30% power. Sonication was performed for 30 s followed by a 30 s pause, and the cycle was repeated six times. The lysates were then centrifuged at 12 000 × g for 10 min, and the supernatants were collected. Protein concentrations were determined using a BCA Protein Assay Kit (Pierce, USA, Cat. #23227), and absorbance was measured at 562 nm with a microplate reader (Multiskan FC, Thermo Fisher Scientific, USA).

Protein samples were separated by SDS-PAGE using a Mini-PROTEAN Tetra system (Bio-Rad, USA). Electrophoresis was first performed at 80 V for 30 min and then continued at 120 V until the bromophenol blue dye front reached the bottom of the gel. The separated proteins were transferred onto PVDF membranes (Millipore, USA, Cat. #IPVH00010) using a Trans-Blot Turbo transfer system (Bio-Rad, USA). After transfer, membranes were blocked with 5% BSA (Sigma-Aldrich, USA, Cat. #A2153) for 1 h at room temperature. Primary antibodies against cGAS, STING, IRF3, and TBK1 were diluted 1:1000 and incubated with the membranes overnight at 4 °C. After washing, the membranes were incubated with HRP-conjugated secondary antibodies (Santa Cruz Biotechnology, USA, Cat. #sc-2004; 1:5000) for 1 h at room temperature. Protein bands were then detected using an enhanced chemiluminescence (ECL) substrate (Thermo Fisher Scientific, USA, Cat. #34080), and images were acquired with a ChemiDoc MP imaging system (Bio-Rad, USA).

Total RNA was extracted from treated cells and harvested brain tissues and then reverse-transcribed into cDNA. The mRNA levels of cGAS and downstream STING signaling-related genes were measured by reverse transcription-quantitative polymerase chain reaction (RT-qPCR) using SYBR Green Master Mix (Thermo Fisher Scientific, USA, Cat. #A25742) and gene-specific primers. Quantitative PCR was performed on an ABI 7500 Real-Time PCR System (Applied Biosystems, USA). Each sample was tested in triplicate, and data are presented as the mean ± standard deviation (SD). Relative gene expression was calculated using the 2^−ΔΔCt^ method, with glyceraldehyde-3-phosphate dehydrogenase (GAPDH) serving as the internal control.

### Mitophagy detection

Mitophagosome formation was examined by TEM (JEOL JEM-1400, Japan). Treated cells and brain tissues were fixed with 2.5% glutaraldehyde for 24 h and washed three times with 0.1 M phosphate buffer (pH 7.4) for 10 min each. Samples were then post-fixed with 1% osmium tetroxide (OsO_4_) at 4 °C for 1–2 h, dehydrated through a graded ethanol series of 30%, 50%, 70%, 90%, 95%, and 100% for 10 min each, and further dehydrated in acetone. After dehydration, samples were embedded in epoxy resin and polymerized at 60 °C for 48 h. Ultrathin sections of 70–90 nm were prepared using an ultramicrotome (Leica EM UC7, Germany) and mounted on copper grids. Sections were stained with 2% uranyl acetate and lead citrate for 15 min each. Mitophagosomes were observed under a TEM, and images were acquired for analysis. For each sample, at least five fields of view were examined, and representative images were selected.

### Western blot

Total protein was isolated from treated cells and harvested brain tissues using RIPA lysis buffer (Beyotime, China), followed by quantification with a BCA Protein Quantification Kit (Pierce, USA). The prepared protein samples were separated by SDS-PAGE and subsequently transferred onto PVDF membranes (Millipore, USA). Protein detection was carried out using primary antibodies against TNF-α, IL-1β, LC3, and p62, and the target bands were visualized with an ECL substrate (Thermo Fisher Scientific, USA).

### RT-qPCR

Total RNA was isolated from treated cells and harvested brain tissues using TRIzol reagent (Invitrogen, USA), followed by reverse transcription into cDNA. The mRNA levels of inflammatory cytokines and mitophagy-associated genes were quantified using SYBR Green Master Mix (Thermo Fisher Scientific, USA) on an ABI 7500 Real-Time PCR System (Applied Biosystems, USA). All samples were analyzed in triplicate. Relative gene expression was calculated using the 2^−ΔΔCt^ method and normalized to GAPDH.

### Immunofluorescence staining

The localization and expression of mitophagy-related proteins were observed using anti-LC3 and anti-p62 antibodies (Cell Signaling Technology, USA) with confocal microscopy (Leica TCS SP8, Germany).

Treated cells and brain tissues were initially labeled with MitoTracker Deep Red to visualize mitochondria, followed by fixation for immunofluorescence staining. The samples were then incubated with primary antibodies against LC3 (Cell Signaling Technology, USA, Cat# 3868S; 1:200) or p62 (Cell Signaling Technology, USA, Cat# 5114S; 1:200). Subsequently, Alexa Fluor 488- or Alexa Fluor 555-conjugated secondary antibodies (Invitrogen, USA, Cat# A11001 or A21428; 1:500) were applied. Fluorescence images were captured using a laser scanning confocal microscope (FV1000, Olympus) equipped with a ×40 oil-immersion objective lens (UPlanSApo, numerical aperture 1.3, Olympus). LC3 or p62 fluorescence signals and their colocalization with mitochondrial signals were assessed in both cells and brain tissues. After acquisition, image contrast and brightness were optimized using Photoshop CS4 (Adobe Systems). For each sample, at least five fields of view were examined, and representative images were selected for further analysis. Colocalization was defined as the overlap between LC3- or p62-positive puncta and MitoTracker Deep Red-labeled mitochondria.

## STATISTICAL ANALYSIS

All data were analyzed using GraphPad Prism 8 software, and the results were expressed as mean ± standard deviation (SD). Differences among groups were analyzed by one-way ANOVA followed by Tukey's post hoc test. Survival data were evaluated using Kaplan–Meier survival curves and compared with the log-rank test. Statistical significance was set at *p* < 0.05.

## RESULTS

### cGAS-targeting nanobody CO@OMVs prepared by genetic engineering technology exhibit uniform particle size distribution and smooth spherical or elliptical structures

The cGAS-targeting nanobody CO@OMVs were successfully prepared using genetic engineering technology, with the synthesis and characterization process of OMVs shown schematically in Fig. 1(a). DLS analysis indicated that the CO@OMVs had a uniform particle size distribution, with an average particle size of approximately 40 nm [Fig. 1(b)]. TEM revealed that the OMVs had a smooth surface and a spherical or elliptical structure [Fig. 1(c)]. WB analysis demonstrated that the CO@OMVs successfully expressed the His-tagged nanobody, confirming the successful modification of the OMVs with the nanobody on their surface [Fig. 1(d)]. Specifically, the signal intensity of the His-tag signal detected by WB validated the effective binding and targeted display of the nanobody.

**FIG. 1.**
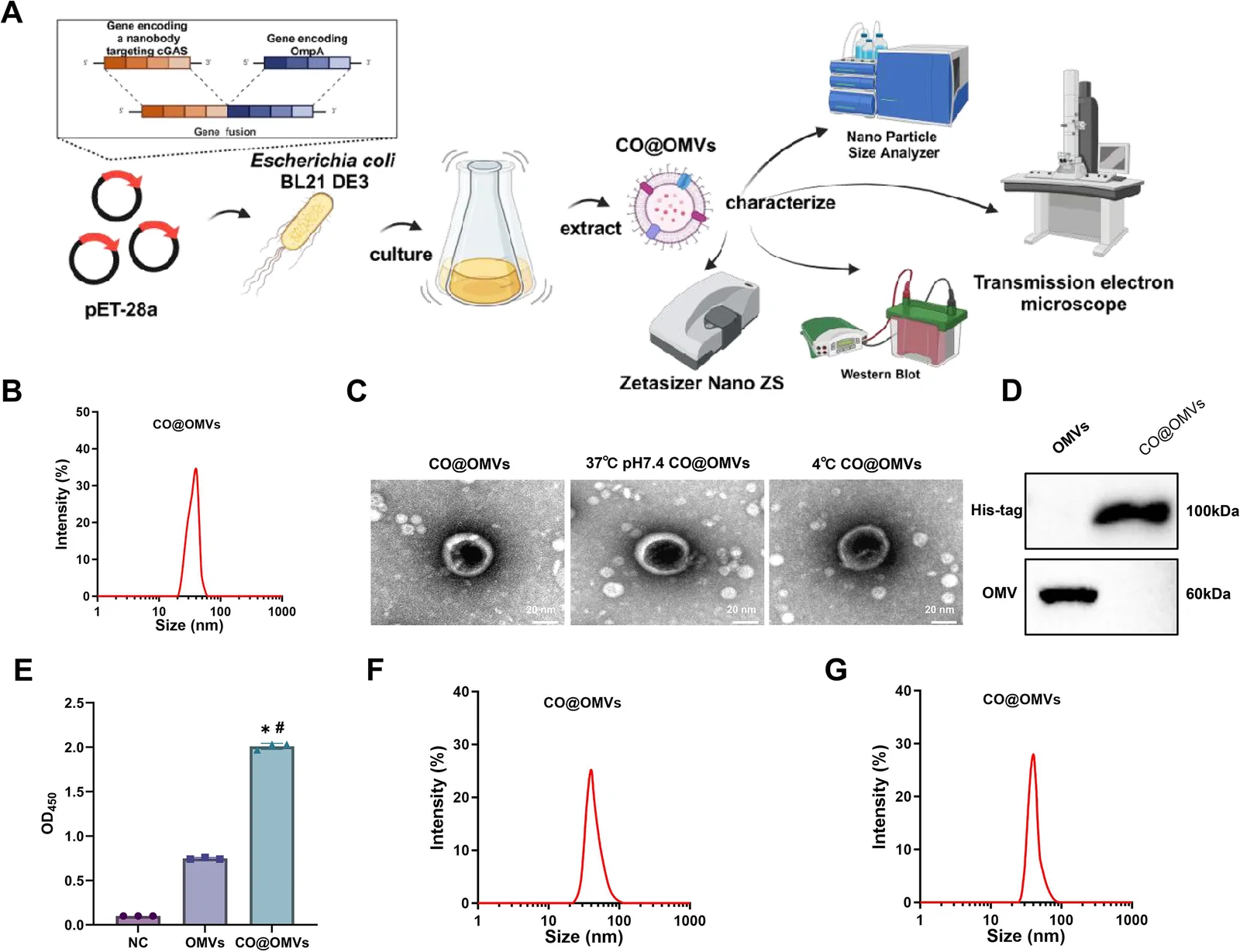
Characterization of particle size distribution and morphology of cGAS-targeting nanobody CO@OMVs. (a) Schematic representation of the synthesis and characterization process of OMV materials; (b) DLS analysis of the nanoparticle size distribution of CO@OMVs; (c) TEM imaging of CO@OMV morphology; (d) Western blot analysis of the His-tagged nanobody modification; (e) ELISA for assessing the binding affinity of CO@OMVs to the cGAS antigen; (f) stability analysis of CO@OMVs at 37 °C and pH 7.4 using DLS and TEM; (g) stability evaluation of CO@OMVs after long-term storage (4 °C, 3 months) using DLS and TEM. * indicates a statistically significant difference compared to the NC group; # indicates a statistically significant difference compared to the OMV group (^*^p < 0.05, ^#^*p <* 0.05).

The binding ability of OMVs to the cGAS antigen was detected using an ELISA. The results showed that CO@OMVs exhibited significantly higher binding affinity for the cGAS antigen than unmodified OMVs [Fig. 1(e)]. Compared to unmodified OMVs, the binding rate of CO@OMVs increased by approximately threefold, demonstrating the effectiveness of the nanobody modification.

In evaluating the stability of OMVs, DLS was used to analyze the particle size changes under different temperature and pH conditions. The results indicated that CO@OMVs remained stable under physiological conditions (37 °C, pH 7.4), with no significant changes in particle size [Fig. 1(f)]. Additionally, after long-term storage (4 °C, 3 months), there were no noticeable changes in the particle size and morphology of the OMVs, indicating good storage stability [Fig. 1(g)].

### Supplementary validation of the inhibitory effects of CO@OMVs on breast cancer cells

To further validate the targeting function of CO@OMVs, *in vitro* cell experiments were conducted. The inhibitory effects of CO@OMVs on breast cancer cells were assessed using the experimental procedure illustrated in Fig. S2(a). CO@OMVs were introduced into the culture medium of 4T1 murine breast cancer cells to assess their effects on tumor cell proliferation, migration, and apoptosis. CO@OMV treatment markedly suppressed 4T1 cell proliferation and migration while promoting apoptotic cell death [Figs. S2(b)–S2(d)]. These findings indicate that cGAS-targeting nanobody CO@OMVs have potential application value in anti-tumor therapy.

### CO@OMVs enhance macrophage activation and phagocytic function through the cGAS–STING pathway

The effects of CO@OMVs on cGAS–STING-associated signaling molecules and macrophage functional activity were further investigated, as illustrated in Fig. 2(a). The expression levels of cGAS, STING, and their downstream signaling molecules, IRF3 and TBK1, were measured in macrophages treated with CO@OMVs using WB and RT-qPCR. The data revealed that these proteins were markedly increased in the CO@OMV-treated group. As shown in Fig. 2(b), cGAS and STING expression was significantly enhanced following CO@OMVs treatment compared with the control group. Consistently, Fig. 2(c) showed that IRF3 and TBK1 expression levels were also notably elevated in macrophages exposed to CO@OMVs. Collectively, these results indicate that CO@OMVs increase the expression of cGAS–STING-related signaling molecules.

**FIG. 2.**
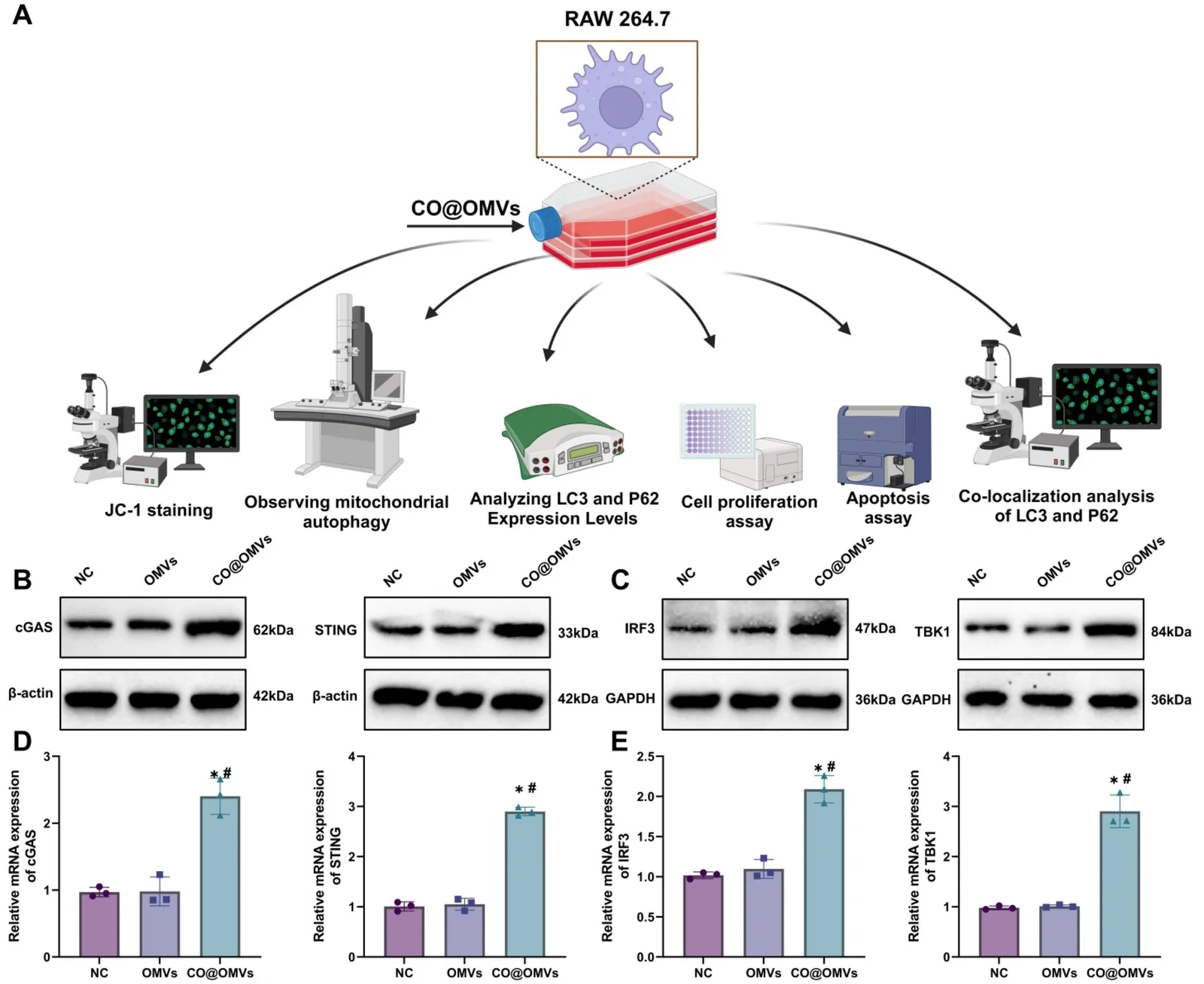
Effects of CO@OMVs on cGAS–STING-associated signaling molecules in macrophages. (a) Experimental flow chart exploring the effects of CO@OMVs on cGAS–STING-associated signaling and macrophage function; (b) Western Blot analysis of the changes in cGAS and STING protein levels in RAW 264.7 macrophages after CO@OMVs treatment; (c) Western Blot analysis of the changes in IRF3 and TBK1 protein levels in RAW 264.7 macrophages after CO@OMVs treatment; (d) RT-qPCR analysis of the changes in cGAS and STING mRNA expression levels in macrophages after CO@OMVs treatment; (e) RT-qPCR analysis of the changes in IRF3 and TBK1 mRNA expression levels in macrophages after CO@OMVs treatment. * indicates a statistically significant difference compared to the NC group; # indicates a statistically significant difference compared to the OMV group (^*^p < 0.05, ^#^*p <* 0.05).

To further verify the WB findings, RT-qPCR analysis was performed to determine the mRNA expression of cGAS, STING, and their downstream signaling molecules, IRF3 and TBK1. As presented in Figs. 2(d) and 2(e), the transcript levels of these genes were markedly elevated in the CO@OMV-treated group compared with the control group. Specifically, cGAS and STING mRNA expression increased by approximately 2.5-fold and threefold, respectively, whereas IRF3 and TBK1 mRNA levels were elevated by about twofold and 2.8-fold, respectively. These results were in agreement with the WB data and further demonstrated increased transcription of cGAS–STING-related signaling molecules following CO@OMV treatment.

To investigate the relationship between cGAS–STING-associated signaling molecules and macrophage function, we performed a series of functional assays. CO@OMV treatment enhanced macrophage activation and phagocytic activity. Fluorescence microscopy showed increased engulfment of target cells by macrophages in the CO@OMV-treated group compared with the control group [Fig. 3(a)]. In addition, flow cytometry further confirmed a significant elevation in the phagocytic index, suggesting that CO@OMVs effectively strengthened macrophage-mediated phagocytosis [Fig. 3(b)]. ELISA analysis of macrophage culture supernatants showed that CO@OMVs markedly increased TNF-α secretion while reducing IL-6 production [Fig. 3(c)], indicating a cytokine profile associated with altered cGAS–STING-related signaling. Overall, these findings suggest that CO@OMVs regulate macrophage functional activity in association with changes in cGAS–STING-related signaling.

**FIG. 3.**
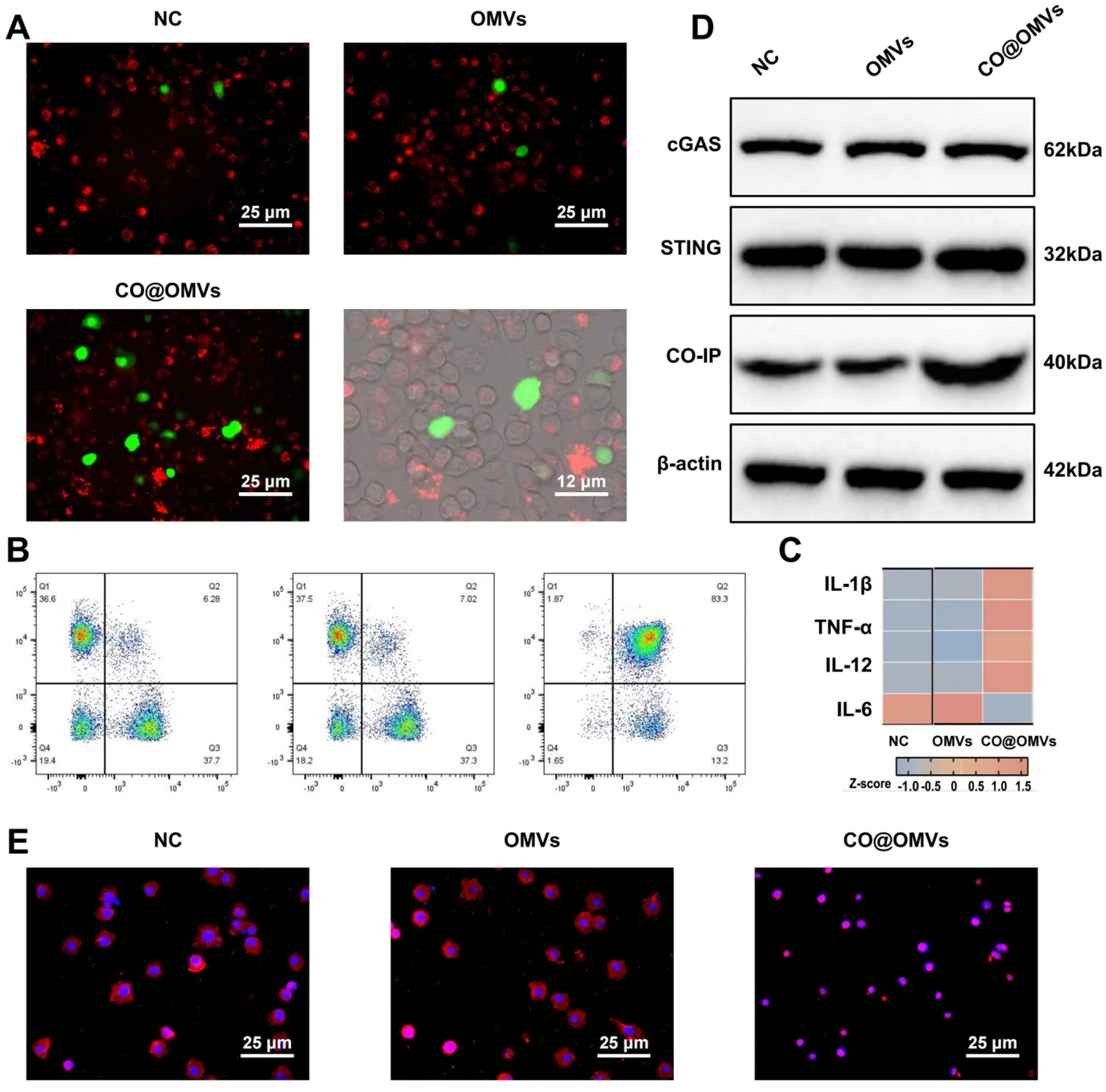
The effects of CO@OMVs on macrophage function and the cGAS–STING signaling pathway. (a) Fluorescence microscopy to observe macrophage phagocytic capacity; (b) flow cytometry analysis of macrophage phagocytic capacity; (c) ELISA to measure the levels of TNF-α and IL-6 in macrophage culture supernatant; (d) immunoprecipitation to detect the interaction between cGAS and STING; (e) immunofluorescence to detect the translocation of STING from the cytoplasm to the nucleus.

To further characterize cGAS–STING-associated signaling changes, immunoprecipitation and immunofluorescence assays were performed. The data showed that CO@OMVs treatment markedly strengthened the association between cGAS and STING in macrophages [Fig. 3(d)], indicating an increased association between cGAS and STING following CO@OMV treatment. Additionally, immunofluorescence analysis showed a marked increase in the translocation of STING from the cytoplasm to the nucleus in the CO@OMV-treated group [Fig. 3(e)], indicating altered STING localization following treatment.

In summary, CO@OMVs were associated with increased cGAS–STING interaction, elevated levels of related signaling molecules, and enhanced macrophage functional activity.

### CO@OMVs significantly induce mitophagy in macrophages and enhance the expression of mitophagy markers

To determine whether CO@OMVs promote mitophagy in macrophages, JC-1 staining, TEM, and Western blot analysis were performed. JC-1 staining showed that CO@OMV-treated macrophages exhibited a significant decrease in mitochondrial membrane potential, suggesting mitochondrial dysfunction and activation of mitochondrial quality-control responses [Fig. 4(a)]. TEM analysis further revealed a markedly increased number of mitophagosomes in CO@OMV-treated macrophages compared with control cells, providing direct morphological evidence of mitophagy induction [Fig. 4(b)]. Consistently, WB analysis showed that CO@OMV treatment significantly upregulated the mitophagy-related proteins LC3-II and p62 [Figs. 4(c) and 4(d)]. These findings, together with the mitochondrial membrane potential changes and ultrastructural evidence, indicate that CO@OMVs effectively induce mitophagy in macrophages and may thereby contribute to macrophage functional regulation.

**FIG. 4.**
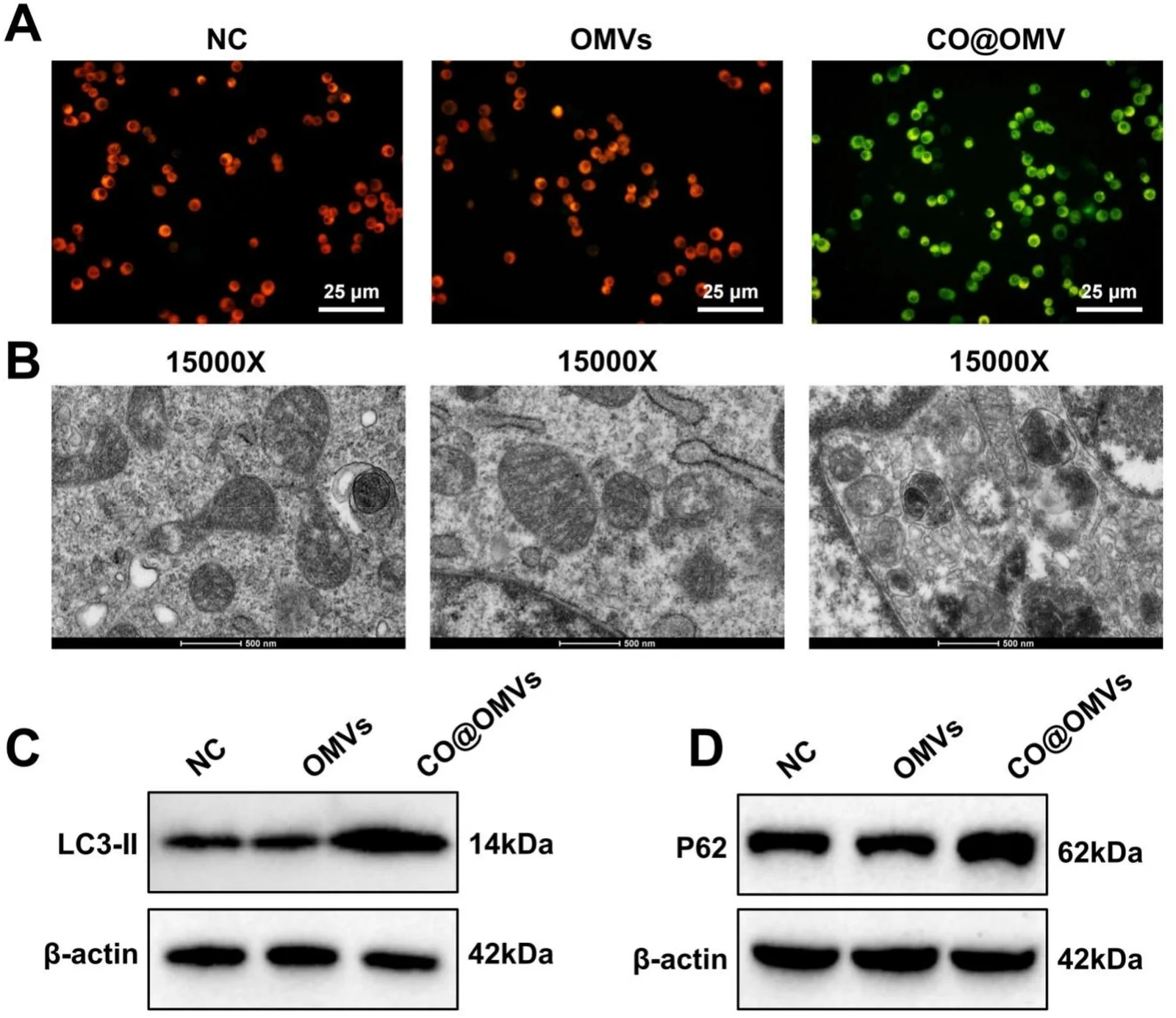
Effects of CO@OMVs on inducing mitophagy in macrophages. (a) JC-1 staining to detect changes in mitochondrial membrane potential in macrophages after CO@OMVs treatment; (b) TEM to observe the number of mitophagosomes in macrophages following CO@OMVs treatment; (c) Western Blot analysis of LC3-II protein expression levels in macrophages after CO@OMVs treatment; (d) Western Blot analysis of p62 protein expression levels in macrophages after CO@OMVs treatment.

### CO@OMVs significantly promote macrophage survival and reduce apoptosis by enhancing mitophagy

The effects of CO@OMVs on macrophage survival, apoptosis, and mitophagy-related mitochondrial localization were evaluated using CCK-8 assays, flow cytometry, and immunofluorescence staining. CCK-8 analysis showed that CO@OMV-treated macrophages exhibited a significantly higher survival rate than control cells, indicating a protective effect of CO@OMVs on macrophage viability [Fig. 5(a)]. Consistently, flow cytometry demonstrated that CO@OMV treatment markedly reduced the overall apoptosis rate, with both early and late apoptotic cell populations notably decreased compared with the control group [Fig. 5(b)]. To further examine whether this protective effect was associated with enhanced mitophagy, immunofluorescence staining was performed to assess the colocalization of LC3- and p62-positive puncta with mitochondria. As shown in Figs. 5(c) and 5(d), CO@OMV-treated macrophages displayed increased overlap between LC3 or p62 signals and mitochondrial signals, suggesting enhanced recruitment of mitophagy-related markers to mitochondria. Together, these results indicate that CO@OMVs promote macrophage survival and reduce apoptosis, at least in part, in association with enhanced mitophagy.

**FIG. 5.**
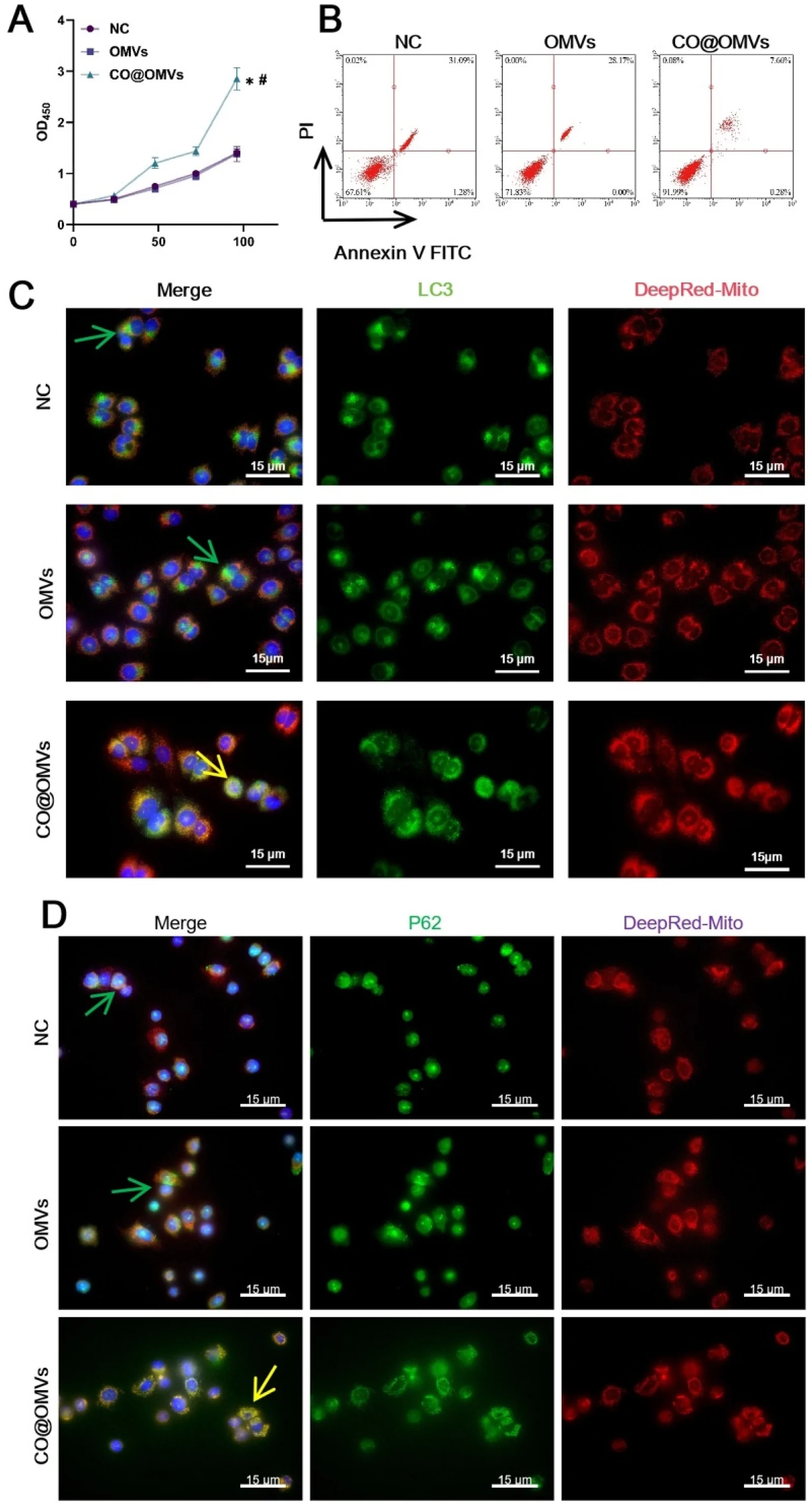
Effects of CO@OMVs on macrophage survival, apoptosis, and mitochondrial colocalization of mitophagy-related proteins. (a) CCK-8 assay to assess macrophage survival after CO@OMVs treatment; (b) flow cytometry to measure the apoptosis rate of macrophages following CO@OMVs treatment; (c) immunofluorescence co-staining of LC3 and MitoTracker Deep Red to detect LC3 colocalization with mitochondria after CO@OMVs treatment (yellow arrows indicate LC3-positive puncta colocalized with mitochondria; green arrows indicate LC3 signals outside mitochondrial regions); (d) immunofluorescence co-staining of p62 and MitoTracker Deep Red to detect p62 colocalization with mitochondria after CO@OMVs treatment (yellow arrows indicate p62-positive puncta colocalized with mitochondria; green arrows indicate p62 signals outside mitochondrial regions). * indicates a statistically significant difference compared to the NC group; # indicates a statistically significant difference compared to the OMV group (^*^p < 0.05, ^#^*p <* 0.05).

### CO@OMVs modulate autophagy-, inflammation-, and immunity-associated genes and proteins in macrophages

To investigate the effects of CO@OMVs on macrophage gene and protein expression profiles, RNA-seq and proteomics analyses were performed. RNA-seq analysis revealed that CO@OMV treatment altered the expression of multiple genes associated with mitophagy, inflammatory regulation, and immune responses. As illustrated in the heatmap in Fig. 6(a), autophagy-related genes, including Beclin1 and Atg1, were markedly upregulated, indicating that CO@OMVs can activate autophagy-related pathways. Additionally, differential gene expression analysis further validated these findings. As shown in Fig. 6(b), the expression levels of autophagy-related genes, such as Beclin1 and Atg1, were significantly higher in the CO@OMV-treated group compared to the control group, indicating effective activation of these genes by CO@OMVs. Proteomic analysis further confirmed the RNA-seq findings and demonstrated substantial changes in autophagy-related proteins and inflammatory mediators in CO@OMV-treated macrophages. As illustrated in Fig. 6(c), the levels of LC3-II, Beclin-1, Atg7, and ULK1 were increased after CO@OMV treatment, accompanied by altered expression of inflammation-related proteins, including TNF-α, NF-κB, IL-1β, and iNOS. Together, these findings indicate that CO@OMVs enhance autophagy-related activity and remodel inflammatory signaling in macrophages by regulating key genes and proteins.

**FIG. 6.**
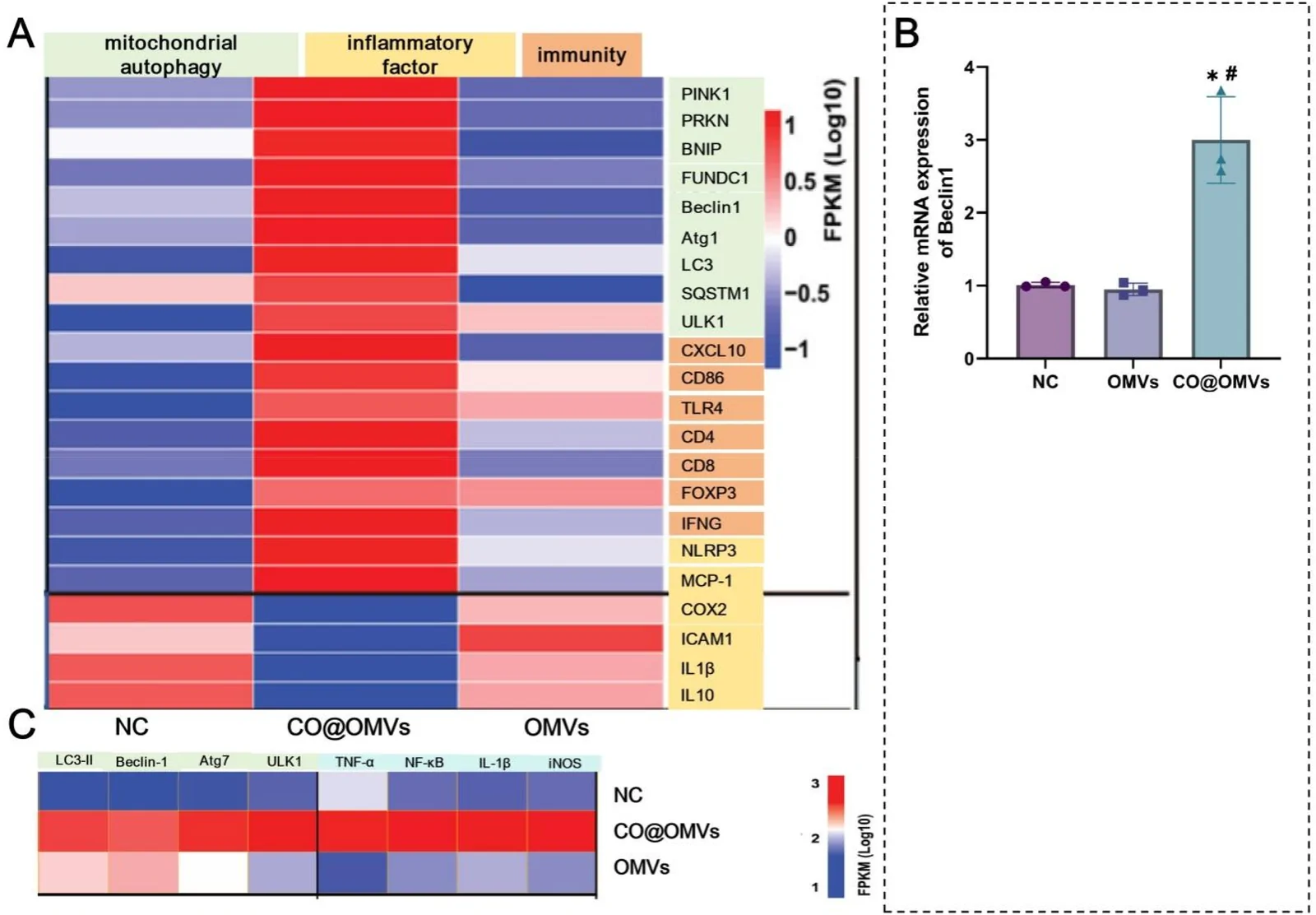
RNA-seq and proteomics analysis reveal the impact of CO@OMVs on gene and protein expression in macrophages. (a) RNA-seq analysis of changes in gene expression related to mitophagy, inflammation, and immunity in macrophages following CO@OMVs treatment; (b) RT-qPCR analysis of the expression changes of Beclin1 and Atg1; (c) proteomics analysis of the expression changes of autophagy-related proteins (LC3-II, Beclin-1, Atg7, and ULK1) and inflammation-related proteins (TNF-α, NF-κB, IL-1β, and iNOS) in macrophages after CO@OMVs treatment. * indicates a statistically significant difference compared to the NC group; # indicates a statistically significant difference compared to the OMV group (^*^p < 0.05, ^#^*p <* 0.05).

### Low-dose CO@OMVs show favorable acute *in vivo* biocompatibility

Serum biochemical analysis was performed to evaluate hepatic and renal toxicity after CO@OMV administration. Low-dose CO@OMVs did not cause obvious hepatic or renal toxicity, whereas high-dose CO@OMVs induced a certain degree of hepatic and renal toxicity, as reflected by changes in ALT, AST, CRE, and BUN levels (Fig. S3).

### CO@OMVs significantly improve cognitive function and motor coordination in a CIRI mouse model

The therapeutic effects of CO@OMVs on cognitive performance and neurobehavioral recovery in the CIRI mouse model were examined using the MWM test and rotarod test, as outlined in Fig. 7(a). In the MWM test, CO@OMV-treated mice showed a significantly shorter escape latency than control mice, indicating improved ability to locate the hidden platform [Fig. 7(b)]. Moreover, the swimming trajectories of CO@OMV-treated mice were more direct and efficient, suggesting improved spatial learning and memory in CIRI mice. Next, motor coordination was evaluated using the rotarod test. As demonstrated in Fig. 7(c), CO@OMV-treated mice remained on the rotating rod for a significantly longer time, indicating a notable improvement in motor coordination. These behavioral results suggest that CO@OMVs promote cognitive and neurobehavioral recovery after CIRI.

**FIG. 7.**
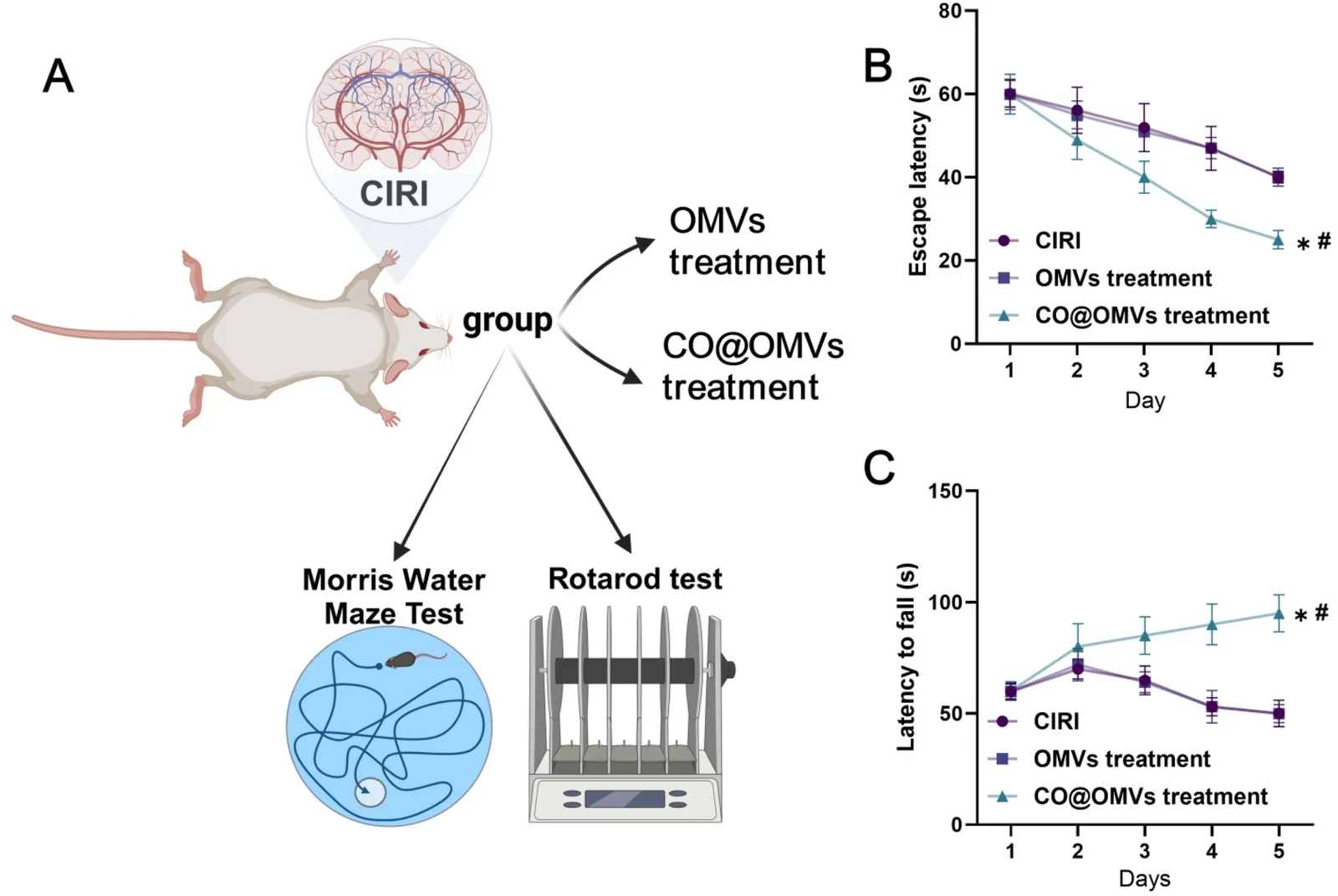
Improvement of cognitive function and neurobehavior in CIRI model mice by CO@OMVs. (a) Schematic diagram of the *in vivo* animal experiment workflow; (b) Morris water maze test assessing the improvement in cognitive function in mice; (c) rotarod test measuring changes in motor coordination in mice. * indicates a statistically significant difference compared to the NC group; # indicates a statistically significant difference compared to the OMV group (^*^p < 0.05, ^#^*p <* 0.05).

### CO@OMVs significantly alleviate inflammation and enhance autophagy-related activity in CIRI model mice to mitigate early brain injury

To evaluate whether CO@OMVs alleviate neuroinflammation and regulate autophagy-related activity in CIRI model mice, H&E staining, IHC, WB, and RT-qPCR analyses were performed. H&E staining showed that CO@OMVs treatment reduced inflammatory cell infiltration and preserved clearer tissue architecture compared with the CIRI and OMVs Control groups [Fig. 8(a)]. Arrowheads and enlarged views indicate scattered inflammatory cells and local tissue morphology. In the control groups, inflammatory cell accumulation, widened tissue spaces, and darker staining were more evident. IHC analysis further showed that TNF-α and IL-1β expression levels were reduced in the brain tissue of CO@OMV-treated mice [Fig. 8(b)]. Consistently, WB and RT-qPCR analyses revealed increased protein and mRNA levels of the autophagy-related markers LC3 and p62 in the CO@OMV-treated group [Fig. 8(c)], suggesting enhanced autophagy-related activity after treatment. Together, these findings indicate that CO@OMVs alleviate neuroinflammation, enhance autophagy-associated responses, and mitigate early brain injury in CIRI model mice.

**FIG. 8.**
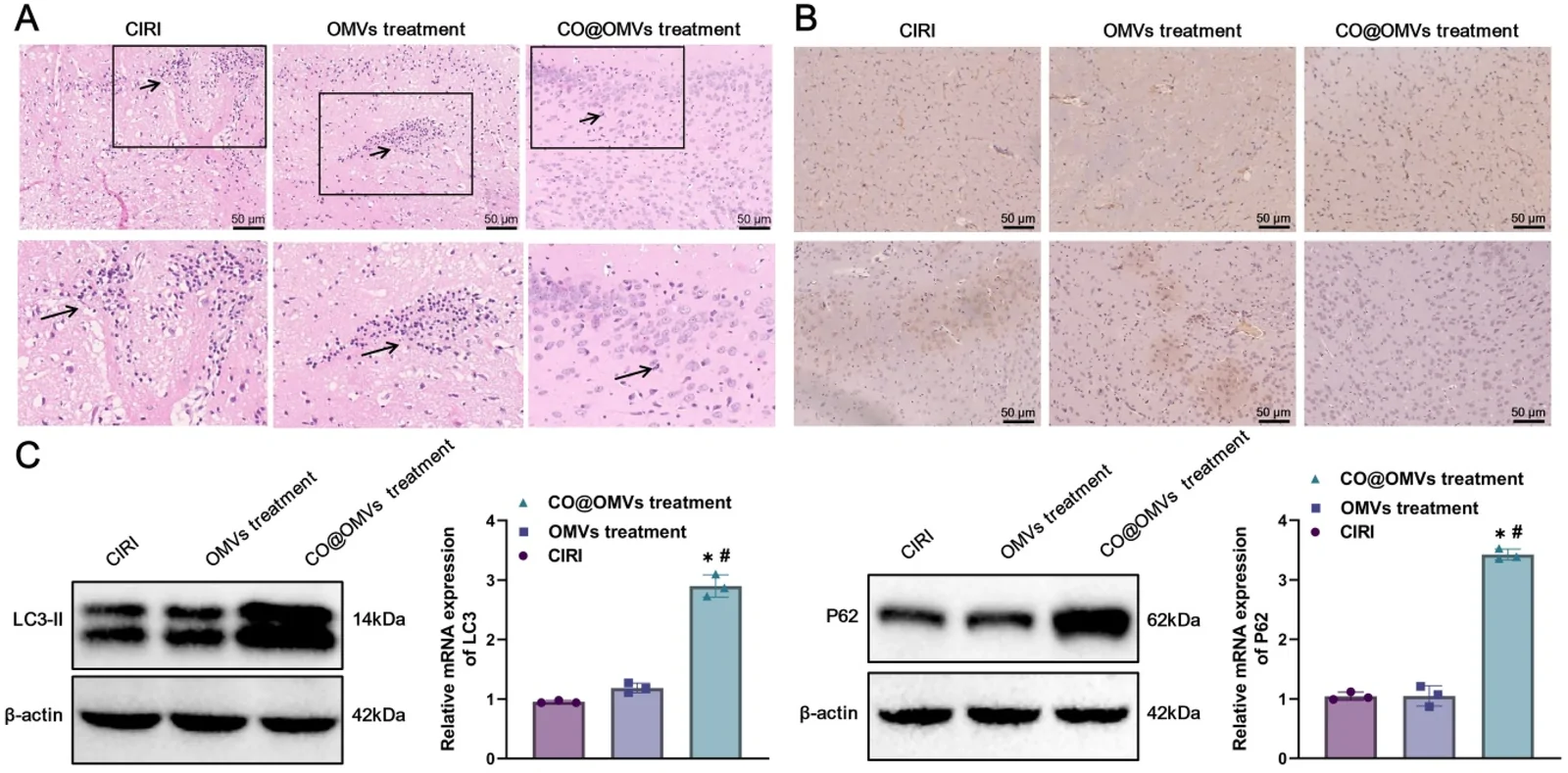
Effects of CO@OMVs on inflammation and autophagy-related activity in brain tissue of CIRI model mice. (a) H&E staining to assess histological injury and inflammatory cell infiltration; arrowheads indicate scattered inflammatory cells, and enlarged views show local tissue morphology; (b) IHC analysis of TNF-α and IL-1β expression; (c) Western blot and RT-qPCR analysis of the expression levels of autophagy-related proteins LC3 and p62, and their mRNA, in brain tissue following CO@OMVs treatment. * indicates a statistically significant difference compared to the CIRI group; # indicates a statistically significant difference compared to the OMVs treatment group (^*^p < 0.05, ^#^*p <* 0.05).

### CO@OMVs significantly induce mitophagy by activating the cGAS–STING pathway

To assess the relationship between CO@OMV-induced mitophagy and cGAS–STING-related signaling, TEM, RT-qPCR, and confocal microscopy analyses were conducted. TEM was first used to examine mitophagosome formation in brain tissues from treated mice. As shown in Fig. 9(a), CO@OMV-treated mice displayed a markedly increased number of mitophagosomes compared with the control group, suggesting that CO@OMVs effectively enhanced mitophagy in brain tissue. RT-qPCR was performed to examine the expression of cGAS, STING, and the downstream signaling molecules IRF3 and TBK1. As shown in Fig. 9(c), CO@OMV treatment significantly upregulated cGAS, STING, IRF3, and TBK1 expression in mouse brain tissues. Confocal microscopy was further used to evaluate cGAS and STING protein colocalization. CO@OMV-treated brain tissues showed markedly enhanced cGAS–STING colocalization compared with controls, indicating increased cGAS–STING colocalization [Fig. 9(b)].

**FIG. 9.**
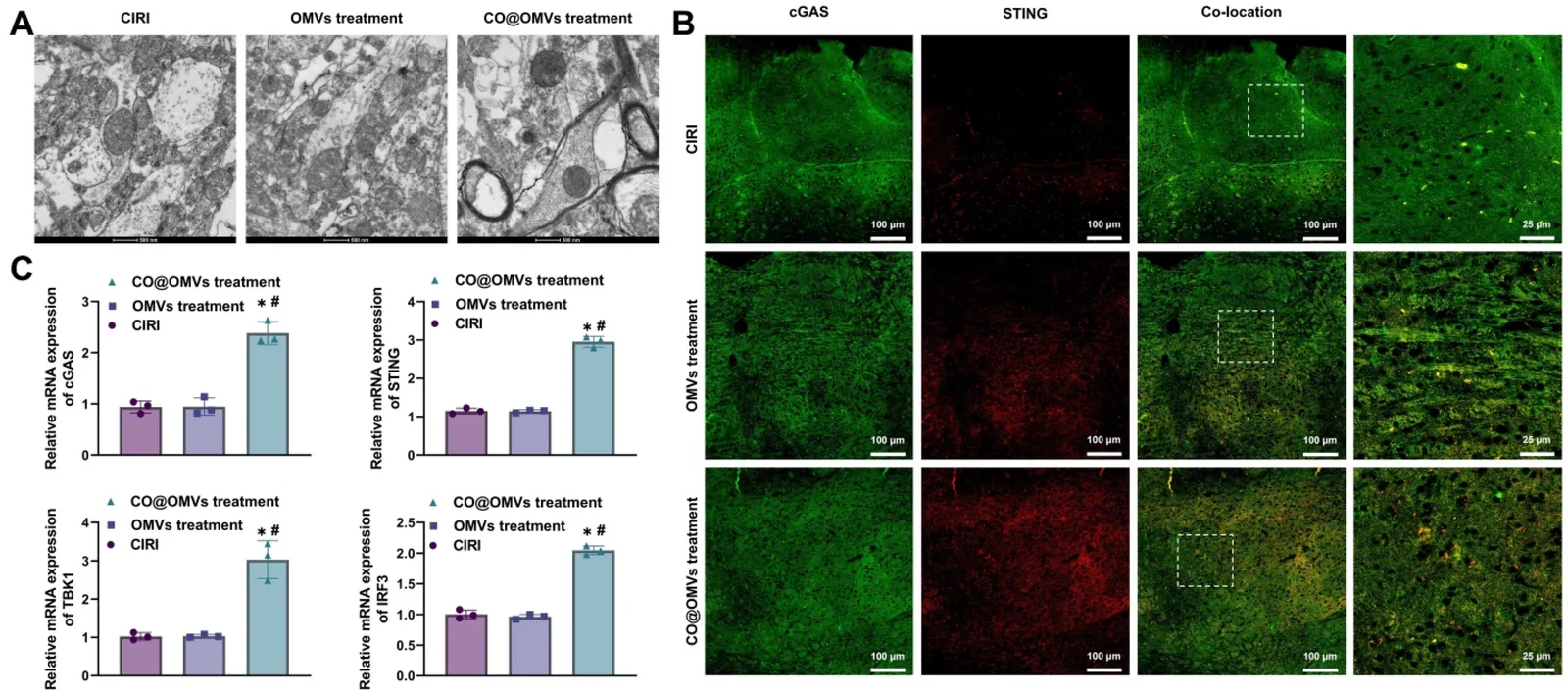
Effects of CO@OMVs on mitophagy and cGAS–STING-associated signaling in CIRI. (a) TEM analysis of changes in mitophagosome quantity; (b) confocal microscopy analysis of the colocalization of cGAS and STING proteins; (c) qPCR analysis of the expression levels of cGAS, STING, and downstream signaling molecules IRF3 and TBK1 in mouse brain tissue. * indicates a statistically significant difference compared to the CIRI group; # indicates a statistically significant difference compared to the OMVs treatment group (^*^p < 0.05, ^#^*p <* 0.05).

In summary, these findings indicate that CO@OMVs promote mitophagy in brain tissue and are associated with changes in cGAS–STING-related signaling.

### The critical role of mitophagy in CO@OMVs treatment of CIRI

A rescue/activation experiment using the mitophagy inhibitor 3-MA and the autophagy activator rapamycin was conducted to validate the essential role of mitophagy in CO@OMVs treatment. The impact of 3-MA or rapamycin on OMV- and CO@OMV-associated mitophagy, inflammation, and apoptosis was assessed. As shown in Fig. 10(a), TEM analysis showed that CO@OMVs increased mitophagosome formation compared with OMVs treatment, whereas 3-MA reduced mitophagosome formation, and rapamycin further promoted mitophagosome accumulation. IHC results showed that 3-MA increased TNF-α and IL-6 staining in both OMV- and CO@OMV-treated tissues, whereas rapamycin attenuated these inflammatory signals [Figs. 10(b) and 10(c)]. Annexin V-FITC/PI flow cytometry further showed that 3-MA increased apoptosis, whereas rapamycin reduced apoptosis, with the CO@OMVs + rapamycin group showing the lowest apoptotic proportion [Fig. 10(d)]. These findings indicate that mitophagy is an important downstream process contributing to the protective effects of CO@OMVs in the CIRI model, providing experimental evidence for further optimizing this treatment strategy.

**FIG. 10.**
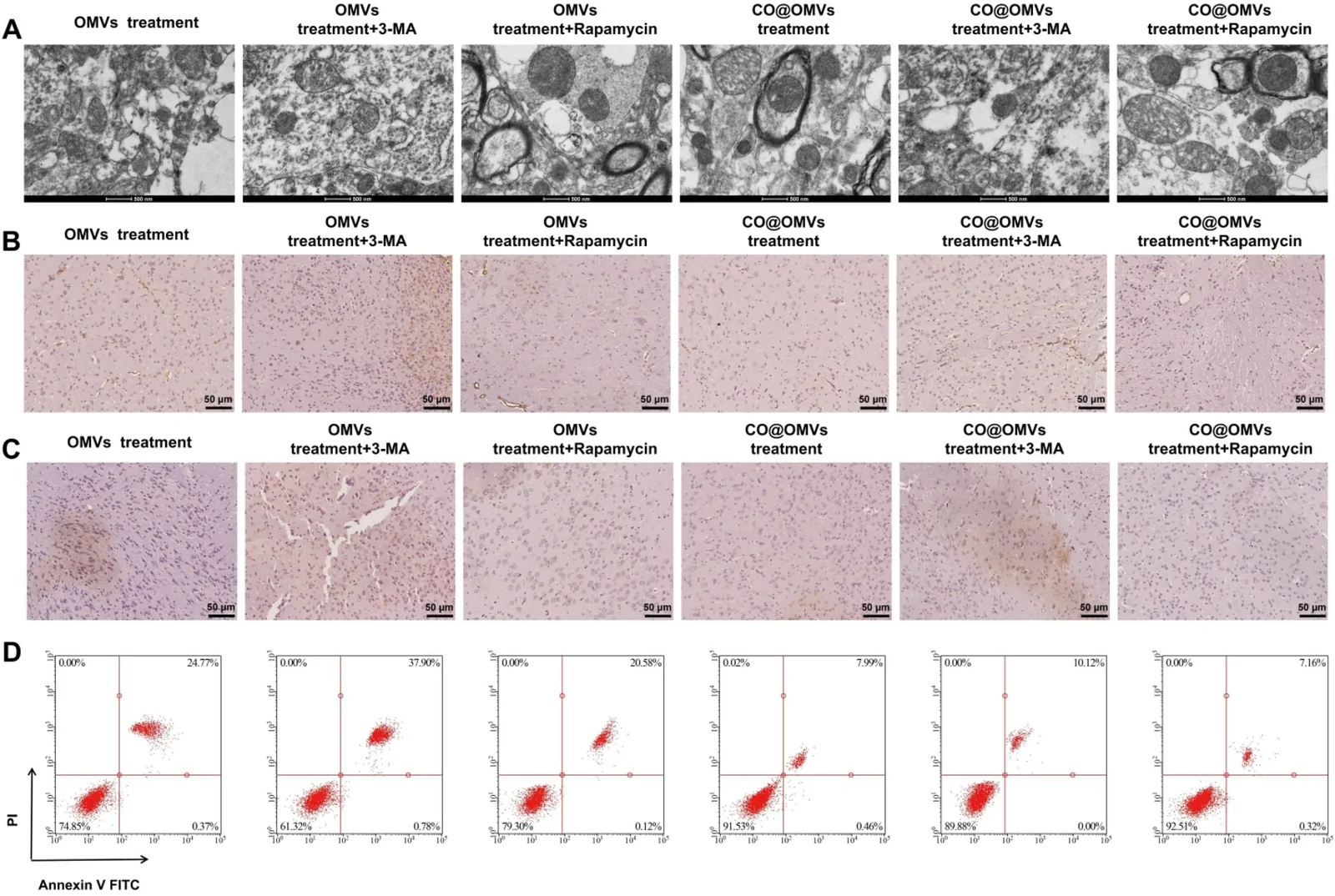
Rescue experiment validating the critical role of mitophagy in CO@OMVs treatment of CIRI. (a) TEM analysis of changes in mitophagosome quantity; (b) IHC analysis of changes in the expression levels of the inflammatory factor TNF-α; (c) IHC analysis of changes in the expression levels of the inflammatory factor IL-6; (d) annexin V-FITC/PI flow cytometry analysis to assess changes in cell apoptosis after 3-MA or rapamycin co-treatment.

## DISCUSSION

This study constructed CO@OMVs that were associated with changes in cGAS–STING-related signaling and induced mitophagy in macrophages, thereby attenuating early CIRI-related injury in the experimental model. Compared with conventional antioxidant or anti-inflammatory approaches, this nanobody-engineered OMV strategy is designed to regulate macrophage-associated innate immune signaling rather than broadly suppress inflammatory responses.^26^ By targeting the cGAS–STING pathway, this study provides evidence that controlled regulation of macrophage activity may represent a potential therapeutic direction for CIRI.

The high targeting specificity of nanobodies endows CO@OMVs with remarkable selectivity in interacting with macrophages, in stark contrast to traditional small-molecule drugs or gene therapy strategies.^27^ Small-molecule drugs often struggle to precisely target specific cell types, leading to nonspecific reactions and increased side effects.^28,29^ While gene therapy has improved targeting capabilities, it still presents certain risks related to safety and stability. In comparison, CO@OMVs leverage the specific binding ability of nanobodies, effectively circumventing these issues, thereby offering a significant advantage in modulating macrophage function. Moreover, OMVs themselves, as bioactive substances, possess inherent immunomodulatory properties, which further support the application of CO@OMVs in the treatment of CIRI.

This study explores the relationship between cGAS–STING-related molecular changes and mitophagy, a topic that has garnered significant attention in the field of immunology in recent years. Previous research has shown that the cGAS–STING pathway is critically involved in antiviral defense and tumor immunity; however, its mechanistic role in CIRI remains incompletely understood.^30^ By applying CO@OMVs, the present study highlights a dual function of cGAS–STING signaling in CIRI. On the one hand, this pathway initiates immune responses by sensing cellular injury signals; on the other hand, it promotes mitophagy, thereby suppressing macrophage-mediated inflammatory responses. These findings expand our understanding of cGAS–STING signaling in ischemic brain injury and provide a potential therapeutic perspective for CIRI intervention.

Multi-omics analysis further revealed that CO@OMV treatment remodeled macrophage gene and protein expression profiles. Compared with previous studies, our findings identified several key molecules and signaling networks associated with mitophagy and inflammatory regulation. These findings offer important insight into the complex pathological processes underlying CIRI, especially the modulation of macrophage function, and may provide a theoretical basis for identifying new therapeutic targets in future studies. By integrating these results with existing evidence, this study establishes a potential connection among these molecules, the cGAS–STING axis, and mitophagy, thereby opening new directions for further investigation.

*In vivo* experiments showed that CO@OMVs improved cognitive and neurobehavioral performance in CIRI mice at the evaluated time point, supporting their protective effect against early brain injury in this model. These outcomes were accompanied by reduced inflammatory marker expression, attenuated histological injury, and enhanced autophagy-related activity in brain tissue. However, because the current *in vivo* evaluation was limited to an early post-injury time point, these findings should be interpreted as preclinical evidence of short-term neuroprotection rather than definitive proof of long-term benefit.

The rescue/activation experiment further supported the contribution of mitophagy to CO@OMVs-mediated protection. Pharmacological inhibition of mitophagy with 3-MA weakened the beneficial effects of CO@OMVs, as reflected by reduced mitophagosome formation, increased inflammatory marker expression, and increased apoptosis. In contrast, rapamycin-mediated autophagy activation further promoted mitophagosome accumulation and was associated with reduced inflammatory marker expression and apoptosis. These findings suggest that mitophagy functions as an important downstream process linking cGAS–STING pathway modulation with the attenuation of early CIRI-related brain injury.

Previous studies have frequently supported cGAS–STING inhibition as a strategy for alleviating CIRI; for example, edaravone and borneol exert neuroprotective effects by suppressing this pathway.^31^ Although cGAS–STING signaling may have context-dependent effects in other disease settings.^32^ persistent cGAS–STING activation has been associated with inflammatory cascades and tissue injury in ischemia-reperfusion injury.^33^ The present data do not establish whether direct activation or inhibition of cGAS–STING accounts for the observed effects of CO@OMVs in CIRI. CO@OMV treatment was associated with altered cGAS–STING-related molecular readouts, enhanced mitophagy, and improved CIRI-related outcomes.

The different inflammatory patterns observed in vitro and in vivo may reflect differences between the experimental systems. *In vitro*, CO@OMV-treated macrophages showed increased TNF-α secretion and decreased IL-6 secretion (Fig. 3), indicating an acute cytokine response in a simplified macrophage culture system. *In vivo*, CO@OMVs treatment reduced local inflammatory markers and improved neurological outcomes in the CIRI model. This divergence may be related to the more complex *in vivo* neurovascular microenvironment, interactions among macrophages, neurons, astrocytes, and endothelial cells, temporal differences between early cell-culture responses and later tissue repair stages, lesion-localized nanocarrier delivery, and differences between soluble cytokine measurements and integrated tissue-level outcomes. Multi-time-point analyses, single-cell sequencing, and cell-specific genetic models will be useful for defining the temporal and cell-type-specific roles of CO@OMVs-regulated cGAS–STING signaling in CIRI.

In summary, cGAS-targeting nanobody CO@OMVs were successfully engineered using gene engineering techniques. CO@OMVs exhibited uniform particle size distribution and smooth spherical or oval-shaped structures. Characterization results confirmed that the CO@OMVs were successfully modified with His-tagged nanobodies, ensuring high affinity for the cGAS antigen. Stability assessments demonstrated that CO@OMVs maintained their integrity under physiological conditions and during long-term storage.

This study developed CO@OMVs that were associated with cGAS–STING-related molecular changes and induced mitophagy in macrophages, thereby alleviating early CIRI-related injury in preclinical models. This strategy highlights the potential role of the cGAS–STING axis in regulating mitophagy and inflammatory responses and introduces a targeted macrophage-modulating approach for CIRI research. Scientifically, these findings deepen the understanding of macrophage-associated innate immune regulation in CIRI. Translationally, CO@OMVs remain a preclinical therapeutic candidate that requires further validation before clinical application.

Several limitations should be acknowledged. First, only acute *in vivo* biocompatibility was evaluated, and the long-term safety, cumulative immune responses, and effects under extended treatment schedules require further study. Second, the animal data were collected at a limited number of time points, which restricts conclusions regarding long-term neurological recovery after CIRI. Third, the study was primarily conducted in mouse models, and whether similar effects can be achieved in other species or humans requires further investigation. Finally, challenges related to delivery route, dose optimization, batch-to-batch consistency, and large-scale production must be addressed before translational application can be considered.

Future studies should further evaluate the application of CO@OMVs in large-scale preclinical experiments and subsequent clinical investigations to comprehensively confirm their safety, efficacy, and therapeutic potential under different pathological conditions. In addition, the design of CO@OMVs could be further optimized by improving targeting efficiency, minimizing potential adverse effects, and integrating them with other therapeutic strategies to enhance overall treatment outcomes. Moreover, future research should extend to additional neural injury models to explore the broader applicability of CO@OMVs in various neurological diseases. With continued technological advances and deeper mechanistic investigations, CO@OMVs may become a promising therapeutic platform and facilitate the clinical translation of nanotechnology for neural injury treatment.

## SUPPLEMENTARY MATERIAL

See the supplementary material for supplementary figures that provide the *in vivo* animal experimental workflow, additional *in vitro* validation of CO@OMV effects on breast cancer cells, and *in vivo* biocompatibility assessment of CO@OMVs.

## Data Availability

The data that support the findings of this study are available from the corresponding author upon reasonable request.

## NOMENCLATURE

cGAS: Cyclic GMP-AMP synthase
CIRI: Cerebral ischemia-reperfusion injury
DLS: Dynamic light scattering
ELISA: Enzyme-linked immunosorbent assay
FBS: Fetal bovine serum
HPLC: High-performance liquid chromatography
H&E: Hematoxylin and eosin
IHC: Immunohistochemistry
IPA: Ingenuity pathway analysis
MCAO: Middle cerebral artery occlusion
OMVs: Outer membrane vesicles
PDI: Polydispersity index
RNA-seq: RNA sequencing
STING: Stimulator of interferon genes
TEM: Transmission electron microscopy
